# Body Mass Index and Retinopathy in Type 1 Diabetic Patients

**DOI:** 10.1155/2014/387919

**Published:** 2014-02-18

**Authors:** Snježana Kaštelan, Jasminka Salopek Rabatić, Martina Tomić, Antonela Gverović Antunica, Spomenka Ljubić, Helena Kaštelan, Branko Novak, Darko Orešković

**Affiliations:** ^1^Department of Ophthalmology, Clinical Hospital Dubrava, Avenija Gojka Šuška 6, 10000 Zagreb, Croatia; ^2^Department of Ophthalmology, University Clinic Vuk Vrhovac, Clinical Hospital Merkur, Zajčeva 19, 10000 Zagreb, Croatia; ^3^Department of Ophthalmology, General Hospital Dubrovnik, Dr. Roka Mišetića 2, 20000 Dubrovnik, Croatia; ^4^Department of Endocrinology and Metabolic Diseases, University Clinic Vuk Vrhovac, Clinical Hospital Merkur, Zajčeva 19, 10000 Zagreb, Croatia; ^5^University of Zagreb, School of Medicine, Šalata 3b, 10000 Zagreb, Croatia

## Abstract

*Aim*. To investigate whether body mass index (BMI) independently or in correlation with other risk factors is associated with diabetic retinopathy (DR) progression. *Methods.* The study included 176 patients with type 1 diabetes divided into three groups according to DR status: group 1 (no retinopathy; *n* = 86), group 2 (mild/moderate nonproliferative DR; *n* = 33), and group 3 (severe/very severe NPDR or proliferative DR; *n* = 57). *Results*. A significant deterioration of HbA_1_c, an increase in total cholesterol, systolic, diastolic blood pressure, and diabetic nephropathy with the progression of retinopathy were found. DR progression was correlated with diabetes duration, HbA_1_c, hypertension, total cholesterol, and the presence of nephropathy. In patients without nephropathy, statistical analyses showed that progression of retinopathy increased significantly with higher BMI (gr. 1: 24.03 ± 3.52, gr. 2: 25.36 ± 3.44, gr. 3: 26.93 ± 3.24; *P* < 0.01). A positive correlation between BMI and a significant deterioration of HbA_1_c, an increase in cholesterol, triglycerides, and hypertension was observed. *Conclusion*. BMI in correlation with HbA_1_c, cholesterol, and hypertension appears to be associated with the progression of DR in type 1 diabetic patients without nephropathy. However, additional studies are required to investigate the pathogenic role of obesity and weight loss in retinal diabetic complications particularly relating to nephropathy.

## 1. Introduction 

Diabetes mellitus is the most frequent endocrine disease in developed countries estimated to have affected 366 million people worldwide and is expected to nearly double by 2030. This growing trend is observed for both type 2 and type 1 diabetes where type 1 accounts for 5–10% of the total cases and its increased incidence is seen across the world in various population studies with the range between 2% and 5% [1–4]. Type 1 diabetes is an autoimmune disease mediated by a combination of genetic and environmental triggers, however, its increasing incidence worldwide cannot be explained by genetic factor alone. Recent studies suggest an alternative possible cause of this epidemic, namely, the role of infections, early childhood diet, environmental pollutants, insulin resistance, and obesity [4].

The global increase of diabetes has a significant impact on the prevalence of diabetic complications among which diabetic retinopathy (DR) takes an important place [5, 6]. DR is a leading cause of acquired blindness in working-age adults and has been estimated to represent 12% of blindness in developed countries [7, 8]. The prevalence of retinopathy increases with the duration of diabetes and is related to hyperglycemia, hypertension, hyperlipidemia, pregnancy, nephropathy, and anemia with genetic factors also having a specific role [9–17]. DR is characterized by the loss of pericytes, hypertrophy of the basement membrane, microaneurysm formation, increased vascular permeability, capillary occlusions, neovascularization, and fibrovascular proliferation. The predominant cause of visual loss in diabetic patients results primarily from intraocular angiogenesis (proliferative diabetic retinopathy, PDR) and leakage of the retinal vessels (diabetic macular edema, DME) [7, 13].

Since DR has become a main cause of vision loss and blindness worldwide intense focus on the early prevention of DR and the benefit of controlling modifiable risk factors has become increasingly important. Although numerous studies have confirmed the role and importance of established risk factors for the development and progression of DR [9, 10], the evidence from new recent trials have nonetheless shown that risk reduction for DR with better glucose and blood pressure management has its limits [18, 19]. Thus, a better comprehension of the role of other modifiable risk factors including obesity in the development of DR becomes even more valuable. The evidence supporting a relationship between high body mass index (BMI) and increased risk of DR is inconclusive [20–33]. Some studies have demonstrated a relationship between obesity or higher BMI and an increased risk of DR [20, 22–29] whilst others have yielded conflicting results [30–33].

Considering that obesity is becoming increasingly prevalent in today's society and since it can be managed by lifestyle intervention particularly exercise, nutrition, and education, studying its effect on diabetic complications has certain logic and benefits. Thus the aim of the present study was to investigate whether obesity independently or in association with other established risk factors influences DR development in type 1 diabetic patients.

## 2. Patients and Methods 

This cross-sectional study was performed in collaboration with the Ophthalmology Departments of three Croatian Hospitals in accordance with the Declaration of Helsinki and approved by the Ethics Committee of each Hospital. The patients included in the study received both written and oral information concerning the study and signed a written informed consent.

### 2.1. Patients

A total of 176 patients with type 1 diabetes were included in the study. All of them were on insulin therapy. Type 1 diabetes was defined according to the American Diabetes Association classification [2]. Patients with immunologic, infectious or inflammatory diseases, and malignancies as well as those taking cytostatics or corticosteroids, pregnant women, and patients with other eye diseases (mature cataract, uveitis, age-related macular degeneration) were not included in the study.

### 2.2. Methods

This study consisted of patients with diabetes who underwent their regular medical and ophthalmological checkups during a one-year period. All patients who met the inclusion criteria were invited to partake in the study and signed the consent form. Blood samples for laboratory analyses were collected between 08:00 and 10:00 am after a 12-hour overnight fast and complete clinical and ophthalmic examinations were done.

#### 2.2.1. Blood Samples

Glycated hemoglobin value (HbA_1_c), total cholesterol, triglycerides, and HDL cholesterol were measured. HbA_1_c was determined by an automated immunoturbidimetric assay (reference values 3.5–5.7%) [34]. Total cholesterol, triglycerides, and HDL cholesterol were measured by the enzymatic colorimetric tests (reference values: total cholesterol <5.00 mmol/L; triglycerides <1.70 mmol/L; HDL cholesterol >1.0 mmol/L) [35, 36].

#### 2.2.2. Anthropometric Parameters

BMI was calculated by dividing weight and height squared (kg/m^2^). A balance-beam scale was used to measure weight, and height was measured using a wall-mounted stadiometer with patients in their underwear and without shoes. Recommended value of BMI among men was considered <23 and among women <22 kg/m^2^ with a normal range being between 18.5 and 24.9 kg/m^2^ [37].

#### 2.2.3. Clinical Parameters

Blood pressure was measured with an ambulatory sphygmomanometric device after a 5 min rest and the mean of three measurements was used. All three measurements were conducted during the same visit. Hypertension was defined as blood pressure >130/80 mmHg or the use of antihypertensive treatment.

#### 2.2.4. Ophthalmologic Examination

Complete eye examination included best corrected visual acuity (BCVA), Goldmann applanation tonometry, slit lamp biomicroscopy of the anterior eye segment, binocular indirect slit lamp fundoscopy, and fundus photography after mydriasis with topically administrated 1% tropicamide and 5% phenylephrine eye drops. Color fundus photographs of two fields: macular field and disc/nasal field of both eyes were taken using a 45° fundus camera (VISUCAM, Zeiss) according to the Europe and Diabetes Study (EURODIAB) retinal photography methodology [38]. Macular field: where the exact centre of the optic disc is laid at the nasal end of the horizontal meridian of the field view. Disc/nasal field: where the optic disc is positioned one disc-diameter in from the temporal edge of the field on the horizontal meridian. The EURODIAB classification scheme was applied since it uses two-field 45° fundus photography and standard photographs to grade retinal lesions [38]. Patients were divided into three groups according to the DR status: group 1 (no retinopathy; *n* = 86), group 2 (mild/moderate nonproliferative DR (NPDR); *n* = 33), and group 3 (severe/very severe NPDR or proliferative diabetic retinopathy (PDR); *n* = 57). The status of the seriously affected eye was the basis on which severity of retinopathy was determined.

#### 2.2.5. Diabetic Nephropathy

Diabetic nephropathy has been categorized and classified into stages based on the values of urinary albumin excretion (UAE): microalbuminuria and macroalbuminuria [39].

### 2.3. Statistical Analyses

Descriptive statistics (*n*, mean ± standard deviation) for all analyzed variables were conducted. Differences in distributions of continuous data were determined by One-Way ANOVA [40]. Scheffe's post hoc test was used. Differences in distributions of categorical data were evaluated by Chi-square test (*χ* ^2^). The relationship between DR, obesity (defined by BMI), and risk factors was analyzed using Pearson's correlation test. Data were analyzed with IBM SPSS software version 12.0 [41]. In all the analyses, *P* value of less than 0.01 and 0.05 were considered statistically significant.

## 3. Results

This study included 176 patients with type 1 diabetes (90 (51.1%) males, and 86 (48.9%) females) with a mean age of 40.03 ± 14.66 years. The mean age at onset was 21.70 ± 13.05 years and the mean duration of diabetes was 18.63 ± 9.27 years. According to the DR status, they were divided into three groups: group 1 (no retinopathy; *n* = 86), group 2 (mild/moderate NPDR; *n* = 33), group 3 (severe/very severe NPDR or PDR; *n* = 57). Incidence of any form of retinopathy in the examined patients (*n* = 176) was 51% of which 10.8% had severe to very severe NPDR and 21.6% had PDR.

Table 1 presents descriptive statistics of basic characteristics of type 1 diabetic patients divided into three groups according to DR status. There was no significant difference in gender between investigated groups. One-Way ANOVA was applied to compare means of age between the three observed groups (45.06 ± 12.56 years versus 37.19 ± 16.62 and 41.40 ± 11.23 years; *P* < 0.05). Scheffe's post hoc test demonstrated only the significant difference between groups 1 and 2 with a test significance of 5%.

Duration of diabetes was significantly different between the three groups (23.03 ± 8.94 and 24.67 ± 7.20 years versus 12.94 ± 6.77 years; *P* < 0.01). Scheffe's post hoc test demonstrated the significant difference between groups 1 and 2, as well as between groups 2 and 3 with a test significance of 1%.

Table 2 presents descriptive statistics of metabolic and clinical parameters of type 1 diabetic patients divided into three groups according to their DR status. We observed a significant deterioration of HbA_1_c (*P* < 0.01) and a significant increase in total cholesterol (*P* < 0.01) as well as systolic (*P* < 0.01) and diastolic blood pressure (*P* < 0.05) with the progression of retinopathy. Scheffe's post hoc test demonstrated the significant difference in mean HbA_1_c value between the groups 1 and 3, as well as between the groups 2 and 3 and in mean total cholesterol value between the groups 1 and 3 with test significance of 1%. The same test demonstrated the significant difference in mean systolic and diastolic blood pressure between the groups 1 and 3 with test significance of 1% for systolic and 5% for diastolic blood pressure.

Patients with NPDR and PDR (groups 2 and 3, resp.) were more often treated with hypolipidemics (*P* < 0.01) and ACE-inhibitors (*P* < 0.01) than patients with no retinopathy (group 1); this was observed by Scheffe's post hoc test with test significance of 1%. We also observed a significant increase in diabetic nephropathy (*P* < 0.01) with the progression of DR.

There was no significant difference in BMI between investigated groups (Table 3). In order to investigate the specific relationship between BMI and DR, patients were additionally divided according to their diabetic nephropathy status into two groups (with/without nephropathy). The group with nephropathy included the patients on dialysis due to diabetic nephropathy whilst the group without nephropathy consisted of patients with normoalbuminuria or microalbuminuria. In type 1 diabetic patients without nephropathy mean BMI was significantly different (*P* < 0.01) (Table 4). Scheffe's post hoc testdemonstrated the significant difference in BMI between the groups 1 and 3 with test significance of 5% (Table 5).

In type 1 diabetic patients with nephropathy, there was no significant difference in BMI between the groups divided according to the DR status (data not shown).

Body mass index was significantly positively correlated with HbA_1_c (*r* = 0.156), total cholesterol (*r* = 0.256), triglycerides (*r* = 0.288), and systolic (*r* = 0.349) and diastolic blood pressure (*r* = 0.282), whereas significant negative correlations between BMI and HDL-cholesterol (*r* = −0.155) as well as diabetic nephropathy (*r* = −0.132) were observed. No correlation between BMI and diabetes duration (*r* = 0.136) was detected. These data are shown in Table 6.

DR was significantly positively correlated (*P* = 0.01) with diabetes duration (*r* = 0.524), HbA_1_c (*r* = 0.375), total cholesterol (*r* = 0.252), and systolic (*r* = 0.175) and diastolic blood pressure (*r* = 0.194) as well as diabetic nephropathy (*r* = 0.761). No correlations between diabetic retinopathy and BMI (*r* = −0.053), triglycerides (*r* = 0.108), and HDL-cholesterol (*r* = −0.029) were observed. These data are shown in Table 7.

In type 1 diabetic patients without nephropathy, DR was significantly positively correlated (*P* = 0.01) with BMI (*r* = 0.247), HbA_1_c (*r* = 0.298), total cholesterol (*r* = 0.378), and systolic (*r* = 0.386) and diastolic blood pressure (*r* = 0.302). In these patients, no correlations between diabetic retinopathy, triglycerides (*r* = 0.160), and HDL-cholesterol (*r* = 0.081), were seen. These data are shown in Table 8.

In type 1 diabetic patients on hemodialysis due to diabetic nephropathy, DR was significantly positively correlated only with HbA_1_c (*r* = 0.499), while significant negative correlation between DR and BMI (*r* = −0.400) was detected. In these patients, no correlations between diabetic retinopathy and total cholesterol (*P* = −0.160), triglycerides (*r* = −0.125), HDL-cholesterol (*r* = −0.046), and systolic (*r* = −0.049) and diastolic blood pressure (*r* = −0.054) were observed. These data are shown in Table 9.

## 4. Discussion 

Although type 1 accounts for only 5–10% of total cases of diabetes, it still represents a significant public health problem due to its rising incidence and prevalence as well as high risk of complications including retinopathy. The incidence of any stage of DR in patients with type 1 diabetes is higher than in type 2 and the advanced forms of DR with visual impairment and blindness usually develop throughout the patients most productive age [1–6]. According to the study, conducted to estimate the global prevalence of DR, age standardized prevalence of any form of DR in type 1 diabetic subjects aged 20–79 years after 10 to 20 years of diabetes duration was 55.55% and 86.22% after more than 20 years. In the same study, the prevalence of PDR after 10 to 20 years was 19.46% and 40.36% after more than 20 years [42]. In our investigation, the mean duration of diabetes was 18.63 ± 9.27 years with the prevalence of any form of retinopathy being 51% while 21.6% of patients had PDR. Furthermore, our results also confirm previous findings that the main risk factors for DR are diabetes duration, prolonged poor glycaemic control, hypertension, and high triglyceride levels [7–10, 15–17]. We found significant differences depending on the duration of diabetes (*P* < 0.01) and the level of glycaemic control (*P* < 0.01) between investigated groups. Advanced stages of DR were present in patients with a longer duration of diabetes and higher values of HbA_1_c. Additionally, a significant difference in the level of systolic (*P* < 0.01) and diastolic blood pressure (*P* < 0.05) as well as total triglyceride levels (*P* < 0.01) between the groups according to their DR status was found.

There are continuous efforts to correlate and explain the dynamic relationship between diabetic retinopathy and nephropathy, the two major microvascular complications of diabetes. Current data suggests that the presence of a preexisting retinopathy or nephropathy may contribute to the development of another, independent of established risk factors for microvascular complications especially in type 1 diabetic patients [43]. Our findings confirm the relationship between DR and nephropathy with obtained results indicating the existence of significant differences for the presence of nephropathy (patients on hemodialysis due to diabetic nephropathy) between the studied groups (*P* < 0.01). The strongest positive correlation of DR with investigated metabolic and clinical parameters was found for the presence of nephropathy (*r* = 0.761). DR was also significantly positively correlated with diabetes duration (*r* = 0.524), total cholesterol (*r* = 0.252), HbA_1_c (*r* = 0.375), systolic (*r* = 0.175), and diastolic blood pressure (*r* = 0.194).

Since current treatment options [7–10, 15–17, 44, 45] fail to entirely eliminate the risk of microvascular diabetic complications, there is a continuing need for the development of new management strategies. Increasing interest is addressed to modifiable risk factors particularly obesity due to its rising incidence and established association with diabetes. To date, the relationship between BMI and DR has been examined in a number of epidemiologic studies giving conflicting results [20–33]. This inconsistency may be partly explained by methodological differences, diversity in study participants, lack of comprehensive anthropometric measurements, inadequate clinical sample size, and particularly racial or ethnic differences [31, 32].

Although several biological theories are proposed, the exact pathophysiological mechanisms supporting the relationship between higher BMI and DR are not entirely clear. Metabolic syndrome, increased oxidative stress, and inflammation due to their association with both obesity and DR have been implied as possible connecting pathogenic mechanisms [8, 14, 20, 46]. Likewise, growing attention has been directed towards the role of vasoproliferative factors in the pathogenesis of DR, whereby their concentrations have been found to be higher in the vitreous of eyes with PDR [47, 48]. Similarly in the serum of obese individuals elevated angiogenic factors have been detected providing additional evidence of the possible bond between obesity and PDR [46, 49]. Obesity and type 1 diabetes are widespread dysmetabolic disorders whose long term complications include severe impairment of the vascular system [50–52]. Several studies have suggested that weight gain may also be involved in the onset as well as long-term progression of type 1 diabetes and may contribute to its rising incidence as confirmed in type 2 [51, 53]. Type 1 diabetes is an inflammatory disease in which its very onset is caused by an inflammatory reaction via lymphocyte-mediated destruction of pancreatic beta cells. This is further followed by a chronic state of low-intense body inflammation episodically aggravated by hyperglycemic fluctuations. Obesity is also a disorder associated with elevated inflammation, oxidative stress, and insulin resistance with growing evidence suggesting that an interrelationship with diabetes could include these mechanisms [52–54]. Epidemiological data have identified hyperlipidemia and hypertension to be connected with obesity as risk factors for DR [28, 50]. In fact, metabolic syndrome encompassing these conditions has also been shown to be associated with retinopathy [55] where many overweight type 1 diabetic patients are difficult to treat and require a relatively high dose of insulin to achieve adequate glycemic control [55, 56]. It is possible that metabolic syndrome and its associated insulin resistance although usually associated with type 2 diabetes may also be a clinical feature for some type 1 diabetic patients [56]. Endothelial dysfunction (ED) as an early indicator of DR is also present in obesity and is characterised by increased levels of adhesion molecules. ED caused by oxidative stress and inflammation processes both connected to diabetes and obesity plays a significant role in the pathogenesis of diabetic arterial wall damage [57–59].

It is generally established that in obese and diabetic individuals, inflammatory and oxidative processes are found to be consistently elevated. The level of inflammatory activation seems to be proportional to severity of obesity in overweight individuals and the quality of glycemic control in type 1 diabetic patients, respectively. Obesity increases the prevalence of several risk factors known to be involved in DR onset and development including inflammatory markers. According to newer concepts, adipose tissue is an active endocrine proinflammatory organ that secretes a large number of bioactive molecules, named adipokines, such as interleukin-6 (IL-6), tumour necrosis factor-*α* (TNF-*α*) leptin, and adiponectin. They regulate body weight homeostasis, influence coagulation, lipid levels, inflammation, oxidative stress, and insulin resistance as well as atherosclerosis and diabetes occurrence. Plasma leptin levels which are elevated in obese individuals and which correlate positively with BMI and insulin resistance [60, 61] have also been related to hypertensive and diabetic retinopathy [61]. Recent findings show that leptin promotes vascular endothelial cell proliferation and angiogenesis in vitro and neovascularisation in vivo [60]. Conversely, adiponectin levels correlate negatively with visceral and subcutaneous fat areas [62, 63] with low adiponectin levels associated with obesity, type 2 diabetes, and insulin resistance [59]. An increased level of adiponectin was found in diabetic and nondiabetic subjects with impaired kidney function as well as in type 1 diabetic patients without complications and particularly in those with diabetic nephropathy [62–65]. In our study, there were no statistically significant differences in BMI between, the investigated groups according to DR status. However, after excluding patients with nephropathy, BMI was significantly higher in those with advanced stages of DR (*P* < 0.01). Moreover, in patients without nephropathy, a statistically positive correlation between DR and BMI was found (*r* = 0.247;  *P* < 0.01) whilst in patients with nephropathy, this correlation was negative (*r* = −0.400; *P* < 0.01).

These obtained findings may be connected to adipokines particularly adiponectin and may be the result of the contradictory effect of obesity and impaired renal function on adiponectin levels [53]. Adiponectin is conversely correlated with BMI, and since kidney dysfunction increases its levels in obese diabetic patients with nephropathy, this mutual influence may have a nullifying effect. In patients without nephropathy, a potential action of adiponectin can be linked to obesity and its impact on oxidative stress and inflammatory markers and therefore DR development may be more pronounced.

Our study has shown that BMI in correlation with poor glycaemic control, hypertension, and dyslipidemia appears to be associated with the progression of DR in type 1 diabetic patients without nephropathy. Several findings indicate the existence of mutual pathogenic mechanisms including inflammation, oxidative stress, and endothelial dysfunction between retinopathy and nephropathy. However, it is not entirely clear in what phase of those complications specific mechanisms dominate or at what stage and to what extent they overlap. Moreover, it is questionable whether these pathogenetic mechanisms impact differently on distinct stages of retinopathy or nephropathy development, whereby a slight modification in action of just one bioactive molecule may cause an entirely different consequential outcome. This is of particular relevance since in chronic kidney disease due to impaired renal biodegradation and elimination, various active protein levels may be increased. Further, it is well established that ED correlates with the progression of renal disorder where it is inactive at the very beginning of the disease yet has an active role in the progression of the glomerulosclerosis causing nephropathy deterioration [66]. Likewise in patients with DR without nephropathy, it is possible that endothelial lesions and dysfunction have less expressed or no influence on retinopathy development.

Obtained results illustrate the value of obesity assessment as a possible modifiable risk factor which may consequently have potential clinical implications on the management of DR. A possible limitation of our study may stem from the cross-sectional design itself in which accurate determination of observed correlation findings may be limited. It would therefore be advantageous to present the course of change in BMI as a very dynamic parameter over time by conducting a prospective study addressing this specific question. Findings showing that BMI is associated with the presence and severity of DR in patients without nephropathy open up implications for further research and intervention in order to elucidate the role of oxidative stress, inflammation, body weight change, and their interaction in the pathogenesis of DR particularly relating to nephropathy. In patients with DR and without nephropathy, the obtained results may also have clinical implications since in these patients, a tight regulation of body weight managed by lifestyle intervention is highly advisable. In this case, alongside glycaemic, hypertension, and lipid control, we may have an additional changeable risk factor which could influence DR development and onset itself. A better and more detailed understanding of all the facets linking obesity and diabetes with the components of the underlying inflammatory and oxidative dysregulation represents the source of future ability for the successful prevention and management of DR.

## Figures and Tables

**Table 1:** Basic characteristics of type 1 diabetic patients (*n* = 176) divided into three groups according to diabetic retinopathy status.

	Group 1 (*n* = 86)	Group 2 (*n* = 33)	Group 3 (*n* = 57)	*P*
Sex (m/f)**	46.51/53.49	54.55/45.45	53.14/43.86	ns
Age (years)*	37.19 ± 16.62	45.06 ± 12.56	41.40 ± 11.23	<0.05
Diabetes duration (years)*	12.94 ± 6.77	23.03 ± 8.94	24.67 ± 7.20	<0.01

*mean ± SD ^∗∗^%; percentage.

Group 1: no retinopathy, group 2: mild/moderate nonproliferative diabetic retinopathy; group 3: severe/very severe nonproliferative diabetic retinopathy or proliferative diabetic retinopathy.

**Table 2:** Metabolic and clinical parameters of type 1 diabetic patients (*n* = 176) divided into three groups according to diabetic retinopathy status.

	Group 1 (*n* = 86)	Group 2 (*n* = 33)	Group 3 (*n* = 57)	*P*
HbA_1_c (%)*	7.25 ± 1.56	7.47 ± 1.44	8.56 ± 1.32	<0.01
Total cholesterol (mmol/L)*	4.74 ± 1.08	5.18 ± 1.01	5.48 ± 1.17	<0.01
Triglycerides (mmol/L)*	1.42 ± 0.77	1.41 ± 0.81	1.66 ± 0.80	ns
HDL-cholesterol (mmol/L)*	1.28 ± 0.41	1.35 ± 0.36	1.28 ± 0.38	ns
Insulin dosage (IU/kg)*	0.71 ± 0.21	0.77 ± 0.16	0.72 ± 0.17	ns
Systolic blood pressure (mmHg)*	127.21 ± 13.89	135.76 ± 20.75	136.40 ± 20.62	<0.01
Diastolic blood pressure (mmHg)*	79.77 ± 10.06	81.82 ± 9.03	84.65 ± 11.66	<0.05
Antihypertensive treatment**	32.56	60.61	57.89	<0.01
Hypolipidemic treatment**	25.58	39.39	54.39	<0.01
Nephropathy**	5.81	24.24	78.95	<0.01

*mean ± SD **%; percentage.

Group 1: no retinopathy, group 2: mild/moderate nonproliferative diabetic retinopathy; group 3: severe/very severe nonproliferative diabetic retinopathy or proliferative diabetic retinopathy; HbA_1_c: glycated hemoglobin value; nephropathy: patients with type 1 diabetes on hemodialysis due to diabetic nephropathy.

**Table 3:** Body mass index (BMI) of type 1 diabetic patients (*n* = 176) divided into three groups according to diabetic retinopathy status.

	Group 1 (*n* = 86)	Group 2 (*n* = 33)	Group 3 (*n* = 57)	*P*
BMI (kg/m^2^)*	24.06 ± 3.48	25.17 ± 3.22	24.43 ± 4.28	ns

*mean ± SD.

Group 1: no retinopathy, group 2: mild/moderate nonproliferative diabetic retinopathy; group 3: severe/very severe nonproliferative diabetic retinopathy or proliferative diabetic retinopathy; BMI: body mass index.

**Table 4:** Body mass index (BMI) of type 1 diabetic patients (*n* = 124) without nephropathy divided into three groups according to diabetic retinopathy status.

	Group 1 (*n* = 81)	Group 2 (*n* = 25)	Group 3 (*n* = 18)	*P*
BMI (kg/m^2^)*	24.03 ± 3.52	25.36 ± 3.44	26.93 ± 3.24	<0.01

*mean ± SD.

Group 1: no retinopathy, group 2: mild/moderate nonproliferative diabetic retinopathy; group 3: severe/very severe nonproliferative diabetic retinopathy or proliferative diabetic retinopathy; BMI: body mass index.

**Table 5:** *Scheffe's post-hoc test*: dependent variable body mass index (BMI) of type 1 diabetic patients (*n* = 124) without nephropathy was divided into three groups according to diabetic retinopathy status.

(I) diab. ret.	(J) diab. ret.	Mean difference (I−J)	Std. error	Sig.	95% Confidence interval	
					Lower bound	Upper bound
Group 1	Group 2	−1.32886	0.75492	0.216	−3.1985	0.5408
	Group 3	−2.89267*	0.89892	0.007	−5.1190	−0.6663

Group 2	Group 1	1.32886	0.75492	0.216	−0.5408	3.1985
	Group 3	−1.56381	1.04668	0.331	−4.1561	1.0285

Group 3	Group 1	2.89267*	0.89892	0.007	0.6663	5.1190
	Group 2	1.56381	1.04668	0.331	−1.0285	4.1561

*The mean difference is significant at the 0.05 level.

**Table 6:** Correlation between body mass index and metabolic and clinical parameters in type 1 diabetic patients (*n* = 176).

	Body mass index (BMI)
	Correlation test*
Diabetes duration (years)	0.136
HbA_1_c (%)	0.156*
Total cholesterol (mmol/L)	0.256**
Triglycerides (mmol/L)	0.288**
HDL-cholesterol (mmol/L)	−0.155**
Systolic blood pressure (mmHg)	0.349**
Diastolic blood pressure (mmHg)	0.282**
Nephropathy	−0.132

**Correlation is significant at the 0.01 level (2-tailed).

*Correlation is significant at the 0.05 level (2-tailed).

HbA_1_c: glycated hemoglobin value; nephropathy: patients with type 1 diabetes on hemodialysis due to diabetic nephropathy.

**Table 7:** Correlation between diabetic retinopathy and diabetes duration, body mass index, and metabolic and clinical parameters in type 1 diabetic patients (*n* = 176).

	Diabetic retinopathy
	Correlation test*
Diabetes duration (years)	0.524**
BMI (kg/m^2^)	−0.053
HbA_1_c (%)	0.375**
Total cholesterol (mmol/L)	0.252**
Triglycerides (mmol/L)	0.108
HDL-cholesterol (mmol/L)	−0.029
Systolic blood pressure (mmHg)	0.175*
Diastolic blood pressure (mmHg)	0.194**
Nephropathy	0.761**

**Correlation is significant at the 0.01 level (2-tailed).

*Correlation is significant at the 0.05 level (2-tailed).

BMI: body mass index; HbA_1_c: glycated hemoglobin value; nephropathy: patients with type 1 diabetes on hemodialysis due to diabetic nephropathy.

**Table 8:** Correlation between diabetic retinopathy and diabetes duration, body mass index, and metabolic and clinical parameters in type 1 diabetic patients without nephropathy (*n* = 124).

	Diabetic retinopathy
	Correlation test*
BMI (kg/m^2^)	0.247**
HbA_1_c (%)	0.298**
Total cholesterol (mmol/L)	0.378**
Triglycerides (mmol/L)	0.160
HDL-cholesterol (mmol/L)	0.081
Systolic blood pressure (mmHg)	0.386**
Diastolic blood pressure (mmHg)	0.302**

**Correlation is significant at the 0.01 level (2-tailed).

*Correlation is significant at the 0.05 level (2-tailed).

BMI: body mass index; HbA_1_c: glycated hemoglobin value.

**Table 9:** Correlation between diabetic retinopathy and diabetes duration, body mass index, and metabolic and clinical parameters in type 1 diabetic patients with nephropathy (*n* = 52).

	Diabetic retinopathy
	Correlation test*
BMI (kg/m^2^)	−0.400**
HbA_1_c (%)	0.499**
Total cholesterol (mmol/L)	−0.160
Triglycerides (mmol/L)	−0.125
HDL-cholesterol (mmol/L)	−0.046
Systolic blood pressure (mmHg)	−0.049
Diastolic blood pressure (mmHg)	−0.054

**Correlation is significant at the 0.01 level (2-tailed). *Correlation is significant at the 0.05 level (2-tailed).

BMI: body mass index; HbA_1_c: glycated hemoglobin value.
